# Differences in Causes, Severity, and Treatment Outcomes Between Women and Men with Chronic Cough

**DOI:** 10.3390/arm94010010

**Published:** 2026-02-09

**Authors:** Aleksandra Marchwińska, Katarzyna Mazurek, Katarzyna Białek-Gosk, Elżbieta M. Grabczak, Olga Truba, Karolina Klimowicz, Marta Dąbrowska

**Affiliations:** 1 Student’s Scientific Group “Alveolus”, Medical University of Warsaw, 02-097 Warsaw, Poland; 2 Department of Internal Medicine, Pulmonary Diseases and Allergy, Medical University of Warsaw, 02-097 Warsaw, Polandmgrabczak@vp.pl (E.M.G.); marta.dabrowska1@wum.edu.pl (M.D.)

**Keywords:** chronic cough, cough management, cough severity

## Abstract

**Highlights:**

**What are the main findings?**

- A chronic cough is a common medical condition that is more prevalent in women.

**What is the implication of the main finding?**

- The results of this study indicate that the female predisposition to cough involves not only a higher female prevalence of chronic coughs but also sex-related differences in the distribution of cough causes and responses to etiology-directed antitussive therapy.

**Abstract:**

A chronic cough, defined as a cough persisting for more than eight weeks in adults, is a common clinical problem with a significant impact on patients’ quality of life. This study compares the etiological spectrum and treatment effectiveness of chronic cough in male and female patients. A retrospective analysis was conducted on a cohort of patients diagnosed in the cough clinic between 2017 and 2021. The response to treatment was assessed based on the reduction in cough severity measured using a 100 mm visual analogue scale (VAS). This study included 231 patients: 164 women (70.9%) and 67 men (29.1%). The median duration of cough was 48 months (IQR 24–120). There were no gender differences in age, BMI, smoking history, cough duration, or severity at the initial visit. Upper airway cough syndrome (UACS) and obstructive sleep apnea (OSA) were diagnosed more frequently in men than in women (UACS: 75% vs. 53%, *p* = 0.002; OSA: 21% vs. 6%, *p* = 0.001). Cough severity significantly decreased in both groups; the median VAS score dropped from 55 to 40 mm in women (*p* < 0.0001) and from 69 to 39 mm in men (*p* = 0.009). The effectiveness of chronic cough treatment, measured by the median reduction in VAS score, was greater in men than in women (32 mm vs. 17.5 mm, *p* = 0.006). These gender-specific differences in cough etiology and treatment response suggest that a “one-size-fits-all” approach may be inadequate.

## 1. Introduction

A cough is an essential reflex with a protective function. However, it is also a symptom of many diseases and one of the most common causes for people to seek a physician’s consultation [1]. Usually, it subsides within a few weeks. Chronic cough is defined as a cough that lasts more than 8 weeks in adults, and it requires extended diagnostics [2]. A chronic cough can have profound effects on the quality of life (QoL), as it is often associated with physical symptoms such as fatigue, throat pain, voice disturbances, urinary incontinence, or syncope [3]. The psychosocial consequences of long-lasting cough are related to depression, frustration, and anxiety [4].

The etiology of chronic cough is diverse. Chronic bronchitis related to current smoking and treatment with ACE inhibitors should be considered at the beginning of the diagnostic path. Next, chronic cough may be a symptom of different chronic lung diseases such as COPD, asthma, bronchiectasis, non-asthmatic eosinophilic bronchitis, lung tumours, and interstitial lung diseases [5]. Among other cough triggers, there are upper airway disorders (such as chronic allergic or non-allergic rhinitis and rhinosinusitis), which are recognized collectively as upper airway cough syndrome (UACS), as well as gastroesophageal reflux (GER) and obstructive sleep apnea [6]. However, cough hypersensitivity may be essential for understanding the real cause of a chronic cough, especially the refractory one [7].

A systematic literature review estimated that chronic cough affects 9.6% of the population worldwide and 12.7% in Europe [8]. Its prevalence is higher in women than in men. In a survey from eleven different cough clinics, two-thirds of the patients diagnosed due to chronic coughs were female [9]. Similarly in a recent Korean Cough Registry, women accounted for 66.9% of the population [10]. The female sex is one of the key risk factors for chronic cough [11]. This may be due to a more sensitive cough reflex and sex-related differences in the central processing of cough sensation in women, as documented by both cough provocation tests and functional brain magnetic resonance imaging [9,12]. The course of the disease may also differ between the sexes, with women tending to cough more often [13]. Next, several aspects of cough-related quality of life—such as embarrassment, frustration, or sleep disorders—are more pronounced in women than in men with chronic cough [14]. Additionally, some complications of intense cough such as stress urinary incontinence are more frequent in women due to differences in anatomy, which may have a negatively impact on their QoL [13].

Taking the above-mentioned inequalities into consideration, we aimed to analyze sex-related differences in severity, causes and treatment effectiveness of chronic cough among patients referred to our cough clinic.

## 2. Materials and Methods

### 2.1. General Study Design

The retrospective analysis of data from patients diagnosed due to chronic cough in the cough clinic at the Department of Internal Medicine, Pulmonary Diseases and Allergy between 2017 and 2021 was performed. This analysis was a part of a larger project on the effectiveness of management of adult patients with chronic cough that was led since 2009. This project was approved by the Institutional Review Board of the Medical University of Warsaw (KB/101/2009).

The primary outcome of the analysis was to identify differences between women and men in cough severity and cough-related quality of life. The secondary outcomes were an evaluation of differences in the spectrum of cough causes and the effectiveness of causal cough treatment.

**Figure 1:**
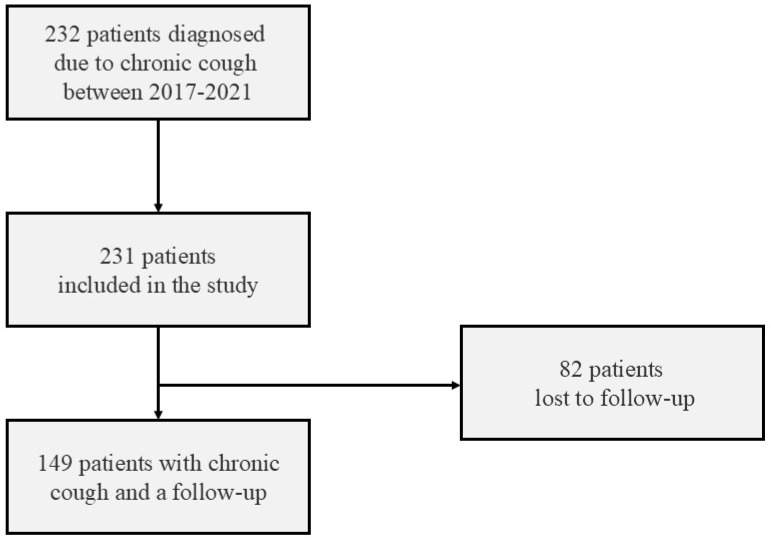
The patients in the study.

All patients referred to the cough clinic due to chronic cough as the main or isolated ailment were asked to participate in this study. The inclusion criteria were as follows: 1. a cough lasting longer than 8 weeks, 2. age above 18 years, 3. normal (or near-normal) chest radiography, and 4. signed informed consent for evaluation of the efficacy of cough treatment. The exclusion criteria were a cough lasting less than 8 weeks, a lack of informed consent and acute airway infection within the last 4 weeks. The data on the efficacy of treatment during 1–3-month follow-ups were available for 149 cases (Figure 1).

### 2.2. Methods

**Figure 2:**
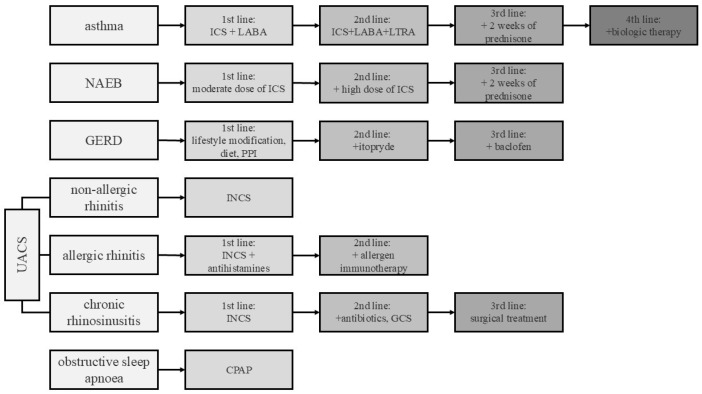
Diagnosis and treatment of patients with chronic cough. *UACS (Upper Airway Cough Syndrome)*, *NAEB (Non-asthmatic Eosinophilic Bronchitis)*, *ICS (Inhaled Corticosteroids)*, *INCS (Intranasal Corticosteroid)*, *GCS (Glicocorticosteroid)*, *LABA (Long-Acting Beta2 Agonist)*, *LTRA (Leukotriene Receptor Antagonist)*, *GERD (Gastroesophageal Reflux Disease)*, and *CPAP (Continuous Positive Airway Pressure)*.

The diagnosis of potential chronic cough causes was performed according to the diagnostic algorithm [15], which was consistent with recent guidelines on the management of chronic cough [16,17]. The treatment was adjusted for the cough diagnosis according to the management draft (Figure 2). The pharmacological intervention was targeted and individualized, based on underlying etiology identified during the initial diagnostic work-up, in accordance with current guidelines [2,5,17]. The subjective severity of the cough was assessed using a 100 mm linear Visual Analogue Scale (VAS), where 0 mm indicated ‘no cough’ and 100 mm represented the ‘worst possible cough’ [18]. Cough-related quality of life was assessed using the Leicester Cough Questionnaire, which comprises 19 items that were grouped into physical, social, and psychological domains. Each item is scored on a 7-point scale, yielding a total score ranging from 3 to 21. The higher the score, the better the quality of life [18].

The response to treatment was assessed based on the reduction in the intensity of cough measured by the VAS or the improvement in QoL measured by the Leicester Cough Questionnaire after 4–12 weeks of therapy at the first control visit [18]. The type of therapy depended on the previous treatment and usually involved escalating the intensity of the etiology-driven therapy to the next step (Figure 2). A good response to the treatment was defined as a decrease in the VAS by at least 30 mm [19] or an increase in the Leicester Cough Questionnaire by at least 1.5 points [20].

### 2.3. Statistical Analysis

Differences between groups were compared using the chi-square test for categorical variables and the Mann–Whitney U test for continuous variables. Statistical significance was set at *p* < 0.05. VAS and LCQ scores before and after the treatment were compared separately for women and men and then analyzed using the Wilcoxon test for dependent variables. The comparison of treatment efficacy between the groups was assessed using the Mann–Whitney test. Statistical analyses were performed using the Medcalc software package (MedCalc^®^ Statistical Software version 23.4.2, Ostend, Belgium)

## 3. Results

Women represented the dominant group in this study, accounting for 70.9% of the 231 patients (164 women vs. 67 men). The median age of all patients was 57 years (IQR: 44.8–67.3), and the median duration of cough was 48 months (IQR: 24–120). The median severity of cough before treatment on the VAS was 61 mm (IQR: 41–78), and the median QoL measured by the LCQ score was 11.26 (IQR: 8.6–13.84). There were no differences between males and females in demographics (age, BMI, or smoking history) or in baseline cough characteristics at the initial visit, including cough duration, character, severity or cough-related QoL (Table 1).

**Figure 3:**
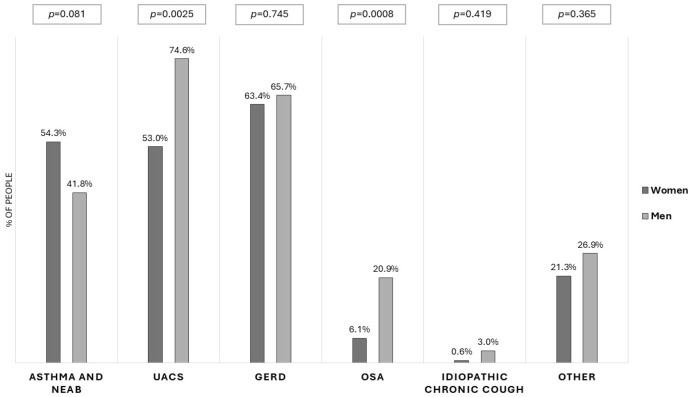
The causes of chronic cough in men and women. *UACS (Upper Airway Cough Syndrome)*, *GERD (Gastroesophageal RefluxDisease)*, and *OSA (Obstructive Sleep Apnea)*.

The majority of our patients had at least two concomitant cough causes; those with a single reason were diagnosed in only 59 subjects (25.5% of patients). Unexplained chronic cough was diagnosed in only three patients. The number of concomitant cough causes was higher in men than in women (2.60 vs. 1.99, *p* = 0.019) (Table 1). The most common chronic cough causes were upper airway diseases, gastroesophageal reflux, and asthma. Upper airway cough syndrome and obstructive sleep apnea were diagnosed more frequently in men than in women (UACS: 75% vs. 53%, *p* =0.0025; obstructive sleep apnea: 21% vs. 6%, *p* = 0.0008) (Figure 3).

After 1–3 months of treatment in both groups, there was a significant reduction in the severity of cough measured by VAS: in women, a reduction in the median VAS value from 55 to 40 mm (*p* < 0.0001) was observed, and in men, it reduced from 69 to 39 mm (*p* = 0.009). An improvement in the quality of life according to the LCQ was also evident; in women there was an increase in the median LCQ score from 11.5 to 13.9 (*p* < 0.0001), while in men, it increased from 10.6 to 14.6 (*p* = 0.0004).

The effectiveness of the chronic cough treatment measured by the median reduction in the VAS was greater in men than in women (32 mm vs. 17.5 mm (*p* = 0.006), respectively). Moreover, there was a difference in the proportion of patients with a good response to treatment measured by the VAS: in the group of men, it was 79.5%, while in women, it was 55.2% (*p* = 0.017). However, the logistic regression model constructed to identify factors associated with a favourable response to chronic cough treatment did not reveal any significant predictors. Neither gender nor the presence of asthma, UACS, or GER emerged as relevant determinants of treatment success.

## 4. Discussion

The problem of female predisposition to chronic cough is an interesting issue, and it is not limited only to its higher prevalence in women than in men. In this study, we did not find differences in cough severity, character, duration, or impact on QoL between men and women, but we demonstrated that among patients referred to our cough clinic, the response to causal antitussive therapy was better in men than in women, even though the number of cough causes was higher in men. In addition, we found a few gender differences in the prevalence of some cough causes such as upper airway diseases and obstructive sleep apnoea, which were more frequent in men than in women.

This observation may provide a useful clue when planning both diagnostic evaluation and the management of patients with chronic cough. It highlights the importance of early diagnosis and treatment of chronic rhinitis or rhinosinusitis, as well as the need for appropriate assessments for sleep apnea in men with chronic cough.

The higher prevalence of chronic cough among women, especially those middle-aged and older, was documented in several studies, mainly those in Europe and North America [9,21,22]. In this study, we also noted a substantial predominance of women (70.9% vs. 29.1%). However, such differences were not documented in some previous studies from China, where chronic cough was found to be equally frequent in men and women [23,24]. This discrepancy might have resulted from the younger patient age in Chinese studies [12]. It seems that the higher the age of the population, the more visible the female predominance among patients with chronic cough. Furthermore, female predominance was also reported among patients with refractory or unexplained chronic cough [25,26,27].

One of the premises to explain the higher female prevalence of cough is the higher cough reflex sensitivity in women than in men, both in patients with chronic cough and in healthy people [28,29]. Furthermore, higher cough sensitivity was observed in older females, which may be due to changes in sex hormones related to menopause (a shortage of estrogen) [30,31,32].

In our study, upper airway cough syndrome was more prevalent in men, which was seldom described before [33]. Although allergic rhinitis is more common than non-allergic rhinitis in the general population in our previous study, non-allergic rhinitis was more frequent than allergic rhinitis in adults with chronic cough [34]. In another study by Cazzoletti et al., the prevalence of non-allergic rhinitis was higher in men than in women in the medium and older groups [35]. Next, male predominance in patients with nasal septum deviation and chronic rhinosinusitis was also recognized, especially in the Asian population [36,37].

In this study, a lower prevalence of obstructive sleep apnea was documented in women with CC, which is consistent with earlier observations. Previous epidemiologic studies showed that obstructive sleep apnea was more prevalent in men than in women [38], while it increased in postmenopausal women [39].

Interestingly, we found a lower response to antitussive therapy in women. Although refractory chronic cough is more common in women than in men [7,24] we found very little real-life data documenting a worse response to cough therapies in females. The gender discrepancies may result not only from more prevalent cough hypersensitivity in women but also from differences in cough drugs, prescription status, or persistence of therapies between men and women [40]. However, when analyzing the logistic regression model, gender was not identified as a relevant predictor of treatment response in our chronic cough population. This may result from the relatively small number of patients in this study; the heterogeneity of cough etiologies, which often coexist; and the variability of therapeutic approaches. Further, larger prospective studies are needed to address this question.

Our study has some limitations. Firstly, it was a single-centre study among patients referred to the cough clinic and mostly included patients with difficult-to-treat CC. Secondly, it was a retrospective analysis, but all patients were diagnosed according to the same worked-out algorithm. Thirdly, the response to therapy was evaluated only using patient-reported outcomes (VAS and LCQ), as cough monitors were not available in our routine real-life practice. Finally, due to the real-life and retrospective nature of this study, we acknowledge limitations related to the uneven distribution of treatments across different etiologies, the coexistence of multiple cough causes, and the lack of assessment of patients’ adherence to the recommended therapies.

## 5. Conclusions

In conclusion, the results of our study demonstrated several differences between women and men, both in the spectrum of cough etiologies and in the effectiveness of chronic cough therapy, thereby contributing to the ongoing discussion on women’s predisposition to chronic cough. Our findings underscore distinct gender-related patterns among patients with chronic cough. These observations suggest that current gender-neutral diagnostic pathways may be suboptimal and highlight the need for more personalized management strategies.

## Figures and Tables

**Table 1:** Study population and cough characteristics stratified by gender.

	Women n = 164	Men n = 67	*p* Value
DEMOGRAPHIC CHARACTERISTICS			
Age (years)	60 (46–68)	55 (39–67)	0.861
BMI (kg/m^2^)	26.8 (23.8–32.1)	26.8 (24.7–30.3)	0.799
Non-smoker/ex-smoker/smoker	136/24/4	56/9/2	0.500
Atopy	35	22	0.066
BASELINE COUGH CHARACTERISTICS			
Duration of cough (months)	48 (24–120)	51 (36–120)	0.631
Dry/productive cough	82/82	32/35	0.757
Baseline cough severity (VAS)	55 (40–80)	69 (53–77)	0.212
Baseline Quality of life (LCQ)	11.54 (8.8–14.2)	10.55 (8.3–12.5)	0.241
NUMBER OF DIAGNOSES	2.30 2.07 to 2.53	1.99 1.86 to 2.13	0.019

Data is expressed as medians and interquartile ranges or numbers and percentages. BMI—body mass index; VAS—visual analogue scale; LCQ—Leicester Cough Questionnaire.

## Data Availability

The raw data are available from the corresponding author upon reasonable request.
